# PAK1 Silencing Attenuated Proinflammatory Macrophage Activation and Foam Cell Formation by Increasing PPAR*γ* Expression

**DOI:** 10.1155/2021/6957900

**Published:** 2021-09-23

**Authors:** Wen-Lin Cheng, Quan Zhang, Bo Li, Jian-Lei Cao, Lin Jiao, Sheng-Ping Chao, Zhibing Lu, Fang Zhao

**Affiliations:** ^1^Department of Cardiology, Zhongnan Hospital, Wuhan University, Wuhan 430071, China; ^2^Institute of Myocardial Injury and Repair, Wuhan University, Wuhan 430071, China; ^3^Department of Obstetrics and Gynecology, Union Hospital, Tongji Medical College, Huazhong University of Science and Technology, Wuhan 430022, China; ^4^Department of Oral Radiology, School and Hospital of Stomatology, Wuhan University, Wuhan 430060, China

## Abstract

Macrophage polarization in response to environmental cues has emerged as an important event in the development of atherosclerosis. Compelling evidences suggest that P21-activated kinases 1 (PAK1) is involved in a wide variety of diseases. However, the potential role and mechanism of PAK1 in regulation of macrophage polarization remains to be elucidated. Here, we observed that PAK1 showed a dramatically increased expression in M1 macrophages but decreased expression in M2 macrophages by using a well-established *in vitro* model to study heterogeneity of macrophage polarization. Adenovirus-mediated *loss-of-function* approach demonstrated that PAK1 silencing induced an M2 macrophage phenotype-associated gene profiles but repressed the phenotypic markers related to M1 macrophage polarization. Additionally, dramatically decreased foam cell formation was found in PAK1 silencing-induced M2 macrophage activation which was accompanied with alternation of marker account for cholesterol efflux or influx from macrophage foam cells. Moderate results in lipid metabolism and foam cell formation were found in M1 macrophage activation mediated by AdshPAK1. Importantly, we presented mechanistic evidence that PAK1 knockdown promoted the expression of PPAR*γ*, and the effect of macrophage activation regulated by PAK1 silencing was largely reversed when a PPAR*γ* antagonist was utilized. Collectively, these findings reveal that PAK1 is an independent effector of macrophage polarization at least partially attributed to regulation of PPAR*γ* expression, which suggested PAK1-PPAR*γ* axis as a novel therapeutic strategy in atherosclerosis management.

## 1. Introduction

Macrophages play crucial roles in regulation of inflammation, innate immunity, and adaptive immunity that the pathophysiology processes induced by macrophage are widely involved in a broad spectrum of acute and chronic inflammatory diseases [1–3]. It is well accepted that macrophages are characterized as remarkable diversity and plasticity which can plasticize to different phenotypes after integrating various environmental signals. Administrated with integration of interferon-*γ* (IFN-*γ*) and Toll-like receptor 4 (TLR4) ligand lipopolysaccharide (LPS), macrophages turns to a classical proinflammatory macrophage phenotype (M1) that regulate inflammatory response by degradation of basement membrane by secretion of extracellular matrix metalloproteinases (MMPs), production of cytokines, and chemokines. On the contrary, macrophages undergo alternative activation (M2) change upon IL-4 and IL-13 stimulation that are associated with resolution of inflammation and tissue repair mediated by secretion of anti-inflammatory cytokines [4–7]. In the past dozens of years, a spectrum of activation programs and underlying molecular mechanisms in M1 and M2 polarized macrophage field have been deeply investigated [8, 9], especially in the development of atherosclerosis in which macrophages are important sources and targets of inflammatory mediators [10]. Accumulative evidences have indicated that a continuum of M1 and M2 macrophages can be expressed in both mouse and human atherosclerotic lesions [11], which, respectively, accelerate or attenuate foam cell formation and atherosclerotic lesion formation [5, 12]. The distinct function of M1 and M2 macrophage polarization implicated in atherogenesis prompts us to explore important regulators and underlying molecular mechanisms.

The P21-activated kinase (PAK) family belongs to the larger nonreceptor serine/threonine protein kinase family with six members (PAK1-6) and initially serves as important effectors for Rho GTPases [13]. Among the PAK family proteins, PAK1 plays a crucial role in a wide variety of diseases, including cancers, inflammation, viral infection, malaria, immunosuppression, ageing, and diabetes [14–18]. Increasing evidences have demonstrated that PAK1 participates in cardiovascular diseases by engaging different signaling pathways [19]. PAK1 acts as a regulator of ion channels and contractile proteins which can prevent arrhythmias by modifying Ca^2+^ homoeostasis in myocytes [20]. *In vitro* and *in vivo* studies demonstrate that PAK1 phosphorylation protects the heart from pressure overload-induced hypertrophy through the JNK/NFAT signaling pathway in response to various hypertrophic stresses [21]. A more recent study demonstrates that PAK1 is a novel therapeutic target for the treatment of ischemia-reperfusion (I/R) injury, as suggested by the evidences that PAK1 deficiency leads to phosphorylation of myofilament proteins and subsequently impedes the recovery of cardiac function after I/R [22]. However, supporting data for the potential functional involvement of PAK1 in macrophage polarization are scarce.

In the present study, we observed that PAK1 expression was positively related with M1 macrophages but negatively associated with M2 macrophages. In functional studies, we demonstrated that PAK1 silencing shaped macrophage towards to anti-inflammatory M2 macrophage, and it mediated M2 macrophage activation dramatically decreased foam cell formation by released cellular cholesterol constituents characterized as upregulated ATP-binding cassette transporter A1 (ABCA1) and ATP-binding cassette transporter G1 (ABCG1) expression but downregulated CD36 and scavenger receptor type A (SR-A) expression. In addition, PAK1 inhibition led to a moderate attenuating effect on M1 macrophage activation. Mechanistically, we demonstrated that the aforementioned effects mediated by PAK1 knockdown were reversed upon inhibited peroxisome proliferator-activated receptor *γ* (PPAR*γ*) expression.

## 2. Materials and Methods

### 2.1. Cell Culture and Adenovirus Infection

Peritoneal macrophages (PMs) were isolated from ApoE-deficient mice and harvested followed with peritoneal lavage treatment for 4 days after intraperitoneal injection of 1 ml of 4% thioglycolate. Then, the cells were collected and cultured in conditioned medium constitute with Roswell Park Memorial Institute (RPMI) containing 10% fetal bovine serum and 1% penicillin-streptomycin. Femurs and tibias from ApoE-deficient mice were flushed with Dulbecco's modified eagle medium (DMEM), and isolated bone marrow-derived macrophages (BMDMs) were centrifuged and cultured in RPMI containing 10% fetal bovine serum and MCSF (50 ng/ml). PLVX-shRNA vector was utilized for PAK1-specific short hairpin RNA- (shRNA-) expressing (shPAK1) construction which then generates AdshPAK1 recombinant adenoviral vectors, while adenoviral vector with short hairpin RNA (AdshRNA) as a control group. BMDMs were infected with adenovirus in diluted media for 24 hours at a 100 multiplicity of infection (MOI) of 100 particles per cell. The collected BMDMs were treated with LPS (50 ng/ml) or IL-4 (10 ng/ml) for 24 hours and harvested for mRNA and protein test. The animal protocols were approved by the Animal Care and Use Committee of Zhongnan Hospital of Wuhan University.

### 2.2. RNA Isolation and Quantitative Real-Time PCR

For real time-PCR analysis, total mRNA was extracted from macrophages with a TRIzol reagent (Invitrogen) manage and then reverse transcribed into cDNA using a Transcriptor First-Strand cDNA Synthesis Kit. PCR amplifications were quantified using a QuantStudio 6 Flex System (Life technologies) in accordance with manufacturer's protocol. The mRNA expressions were normalized to GAPDH expression. The primers were showed in Table 1.

### 2.3. Western Blotting

Cultured macrophages were lysed using a RIPA assay buffer, and protein concentrations were determined using a Pierce BCA Protein Assay kit. Five micrograms of protein were separated via sodium dodecylsulphate polyacrylamide gel electrophoresis (SDS-PAGE) and transferred to a polyvinylidene fluoride (PVDF) membrane, which then probed with particular primary antibodies overnight at 4°C. Following incubation with secondary antibodies for 1 hour at room temperature, the signals were visualized using a FluorChem E Imager. The protein expression levels were normalized against GAPDH. The antibodies were showed in Table 2.

### 2.4. Foam Cell Formation

BMDMs infected with AdshPAK1 upon stimulation with LPS or IL-4 were cultured on chamber slides for overnight incubation. To further visualize cholesterol accumulation, the treated macrophages were fixed on cover slips and stimulated with 15ug/ml of oxidation low lipoprotein (Ox-LDL) for additional 24 hours. Macrophages were then fixed with 4% paraformaldehyde in PBS and stained with 0.3% Oil Red O in 60% isopropanol and captured by microscopy.

### 2.5. Immunofluorescence Staining

Macrophages were cultured on cover slips and were fixed with 3.7% formaldehyde and permeabilized with 0.1% Triton X-100 in PBS for 45 min. Subsequently, the slides were blocked in 10% goat serum diluted with PBS for 1 h and incubated overnight with various primary antibodies overnight at 4°C. After rewarming at 37°C for 1 h, the sections then were washed in PBS and incubated with the appropriate secondary antibodies for another 1 h. Images were captured with a fluorescence microscope (Olympus, Tokyo, Japan) using DP2-BSW software and were analyzed with Image-Pro Plus 6.0.

### 2.6. Co-IP

Immunoprecipitation was performed to determine protein-protein interactions. For immunoprecipitation, cells were washed with cold PBS and lysed with lysis buffer containing Protease Inhibitor Cocktail Tablets (AS1005C, ASPEN). After being precleared with immunoglobulin G and Sure Beads™Starter Kit Protein A (#1614813, BIO-RAD), lysates were incubated with the indicated primary antibodies and protein A-agarose at 4°C overnight with gentle shaking. The immunoprecipitated proteins were further washed five times with lysis buffer, boiled with 2× SDS loading buffer, separated with SDS–PAGE, and electrophoretically transferred to PVDF membrane. The membranes were blocked with 5% BSA in Tris-buffered saline containing 0.1% Tween-20 and were immunoreacted with the indicated primary antibodies and secondary antibodies.

### 2.7. Statistical Analysis

All results were presented as the means ± SD. For comparisons between two groups, Student's two-tailed *t*-test was applied. One-way analysis of variance (ANOVA) was applied for comparison of multiple groups. All statistical analyses were performed. The software SPSS, version 22.0, was used for all statistical analyses. *P* values less than 0.05 were considered significant.

## 3. Result

### 3.1. Altered PAK1 Expression in M1 and M2 Macrophage

**Figure 1:**
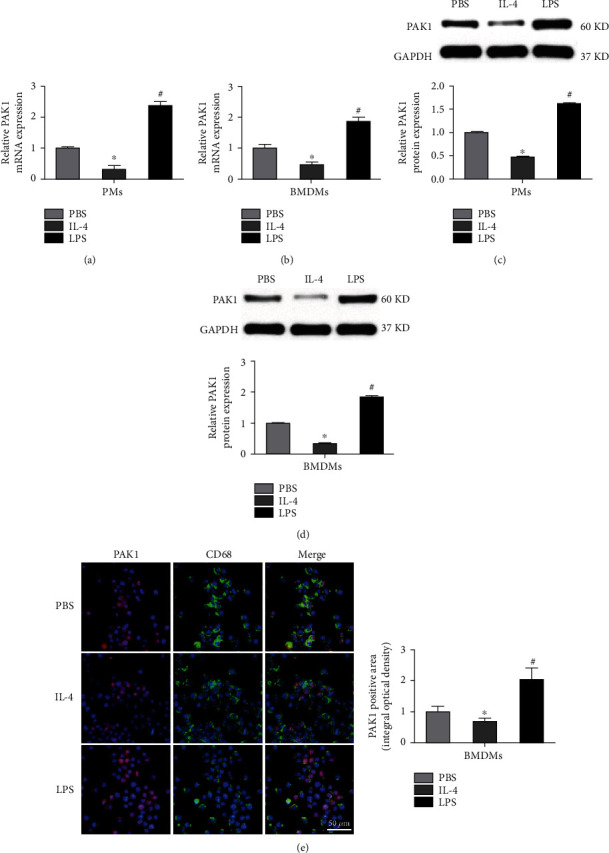
PAK1 expression is induced in M1 but reduced in M2 macrophages. (a, b) PAK1 mRNA levels in PMs and BMDMs subjected to PBS, IL-4, or LPS stimulation for 24 hours. *n* = 3. (c, d) Western blot analysis of PAK1 protein levels in PMs and BMDMs administrated with PBS, IL-4, or LPS for 24 hours. *n* = 3. (e) Representative images of double immunofluorescence staining of BMDMs with an anti-PAK1 antibody (red) and macrophage marker (CD68, green) treated with PBS, IL-4, or LPS for 24 hours. The integral optical density of PAK1 was presented. Scale bar = 50 *μ*m. *n* = 3. ^∗^*P* < 0.05 versus PBS group, ^#^*P* < 0.05 versus IL-4 group. PMs: peritoneal macrophages; BMDMs: bone marrow-derived macrophages; PBS: phosphate-buffered saline; IL-4: interleukin-4; LPS: lipopolysaccharides.

To examine whether PAK1 is involved in regulation of macrophage activation, we first investigated the expression of PAK1 in classically (M1) or alternatively (M2) activated macrophage upon LPS or IL-4 stimulation, respectively. RT-PCR analysis showed that PAK1 mRNA levels were significantly increased in M1 macrophage but decreased in M2 macrophage as assessed with PMs (Figure 1(a)) and BMDM population (Figure 1(b)). As expected, we observed a similar pattern in PAK1 protein expression examined by Western blot analyses (Figures 1(c) and 1(d)). In addition, double immunofluorescence staining for PAK1 and the macrophage-specific marker CD68 in BMDMs showed stronger immunoreactivity of PAK1 in M1 macrophage than that in M2 macrophage (Figure 1(e)). Collectively, these findings suggested a positive relationship between PAK1 and macrophages with the proinflammatory phenotype.

### 3.2. PAK1 Silencing Promoted Alternative M2 Activation

**Figure 2:**
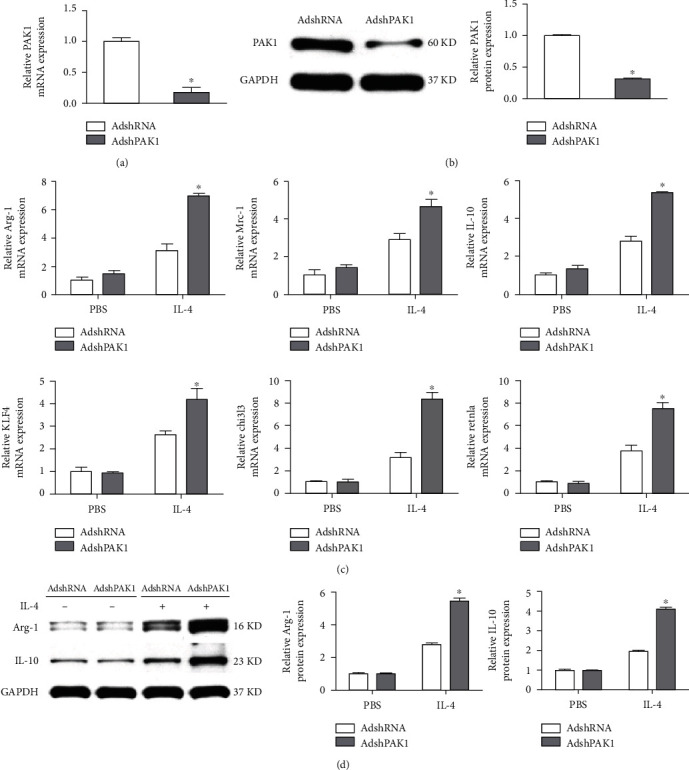
PAK1 promotes M2 polarized macrophages. (a, b) The PAK1 expression in BMDMs infected with AdshPAK1 or AdshRNA. *n* = 3. (c) mRNA expression levels of M2 macrophage markers in BMDMs by PAK1 knockdown with IL-4 stimulation. *n* = 3. (d) Protein expression levels of Arg-1 and IL-10 in BMDMs by PAK1 knockdown with IL-4 stimulation. *n* = 3. ^∗^*P* < 0.05 compared with control group. Arg-1: arginase-1.

Since the notable change of PAK1 expression in M1 and M2 macrophages suggested a possible role for PAK1 in the regulation of macrophage polarization, we next tested the expression of represented markers of M1 or M2 activation in BMDMs regulated by PAK1. A *loss-of-function* study with adenovirus harboring PAK1 short hairpin RNA (AdshPAK1) was performed, and we noticed that PAK1 expression was dramatically decreased in the BMDMs transfected with AdshPAK1 (Figures 2(a) and 2(b)). In response to IL-4 administration, higher mRNA levels of anti-inflammatory M2 makers were found in BMDMs mediated by PAK1 knockdown, including arginase-1 (Arg-1), Mrc-1, interleukin (IL)-10, KLF4, chi3I3, and Retnla, whereas there was no difference under basal conditions in response to PBS stimulation (Figure 2(c)). Furthermore, these changes in the mRNA levels of Arg-1 and IL-10 were recapitulated at the protein level, as determined by Western blot analyses (Figure 2(d)).

### 3.3. PAK1 Knockdown Inhibited Classical M1 Activation

**Figure 3:**
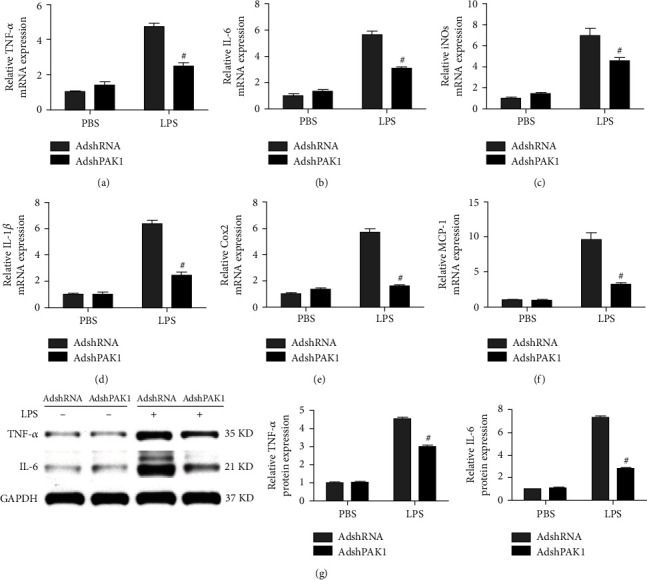
PAK1 attenuates the M1 polarized macrophages. (a–f) RT-PCR analysis of expression levels of M1 macrophage markers in BMDMs by PAK1 knockdown upon LPS treatment. *n* = 3. (g) Western blot analysis of TNF-*α* and IL-6 protein level in BMDMs by PAK1 knockdown upon LPS treatment. *n* = 3. ^#^*P* < 0.05 compared with control group. TNF-*α*: tumor necrosis factor-*α*; iNOs: inducible no synthase.

Next, we evaluated the effect of PAK1 silencing on M1 polarization by testing the prototypical proinflammatory M1 macrophage target genes. In contrast with the observations of AdshPAK1-mediated M2 macrophages, the expression of LPS-induced M1 markers, including the characteristic tumor necrosis factor-*α* (TNF-*α*), IL-6, inducible no synthase (iNOs), IL-1*β*, Cox2, and MCP-1 genes, was significantly decreased by PAK1 knockdown, whereas slight differences were observed in the PBS-treated group (Figures 3(a)–3(f)). As expected, the differences in mRNA expression of TNF-*α* and IL-6 were confirmed by Western blot analyses to be similar for the corresponding proteins (Figure 3(g)).

### 3.4. Decreased Foam Cell Formation Was Mediated by PAK1 Knockdown

**Figure 4:**
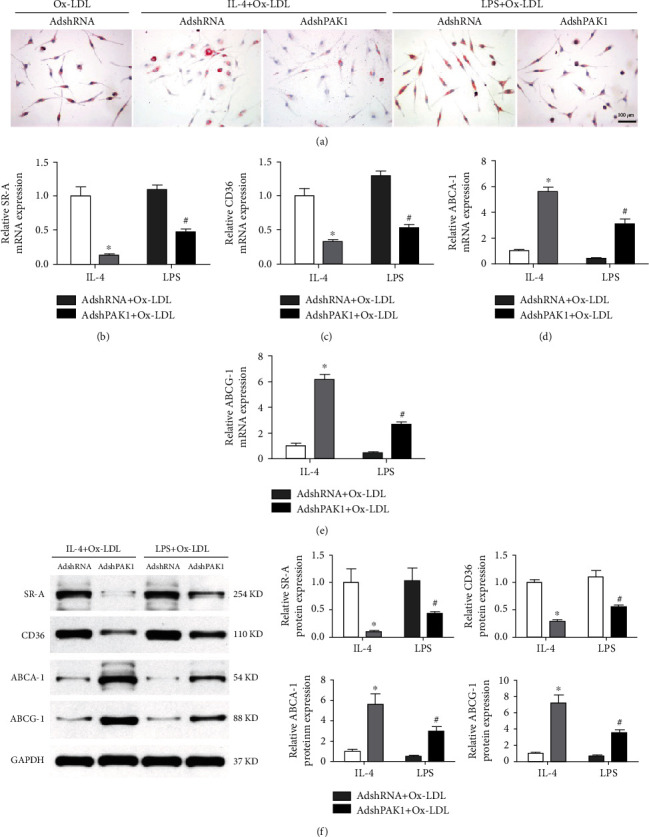
Decreased foam cell formation by PAK1 silencing. (a) Oil-red staining of BMDMs infected with AdshPAK1 or AdshRNA, which previously stimulated with IL-4 or LPS and administrated with Ox-LDL. Scale bar = 100 *μ*m. *n* = 6‐10. (b–f) Alternation of the expression of SR-A and CD36, and ABCA1 and ABCG1 in BMDMs infected with AdshPAK1, which previous stimulated with IL-4 or LPS and administrated with Ox-LDL at mRNA (b–e) and protein (f) levels, respectively. *n* = 3. ^∗^*P* < 0.05 or ^#^*P* < 0.05 compared with control group. SR-A: scavenger receptor type A; ABCA1: ATP-binding cassette transporter A1; ABCG1: ATP-binding cassette transporter G1 (ABCG1).

Foam cells formed by the accumulation of oxidized modified LDL (Ox-LDL) in macrophages play an important role in the development of atherosclerosis, and recent studies have suggested that macrophage polarization may substantially contribute to foam cell precursors. Consistently, we noticed that accumulated foam cells were found in the M1-polarized macrophage population treated with Ox-LDL, but fewer foam cells were found among the M2-polarized macrophages treated with Ox-LDL, as determined by neutral lipid staining with Oil Red O (Figure 4(a)). Importantly, dramatically decreased lipid accumulation was observed in the M2 macrophages activated by AdshPAK1 after treatment with Ox-LDL compared with those treated with AdshRNA, whereas moderately decreased lipid accumulation was found in the M1 macrophages in which activation was mediated by AdshPAK1 (Figure 4(a)). The RT-PCR analysis revealed significantly or moderately decreased marker expression accounted for cholesterol influx (SR-A and CD36) and increased markers related to cholesterol efflux (ABCA1 and ABCG1) in IL-4-induced M2 macrophages and LPS-induced M1 macrophages regulated by PAK1 knockdown, respectively (Figures 4(b)–4(e)). The expression of protein markers related to cholesterol influx and efflux was similar to that observed for the corresponding mRNAs (Figure 4(f)).

### 3.5. PAK1 Silencing Upregulated PPAR*γ* Expression

**Figure 5:**
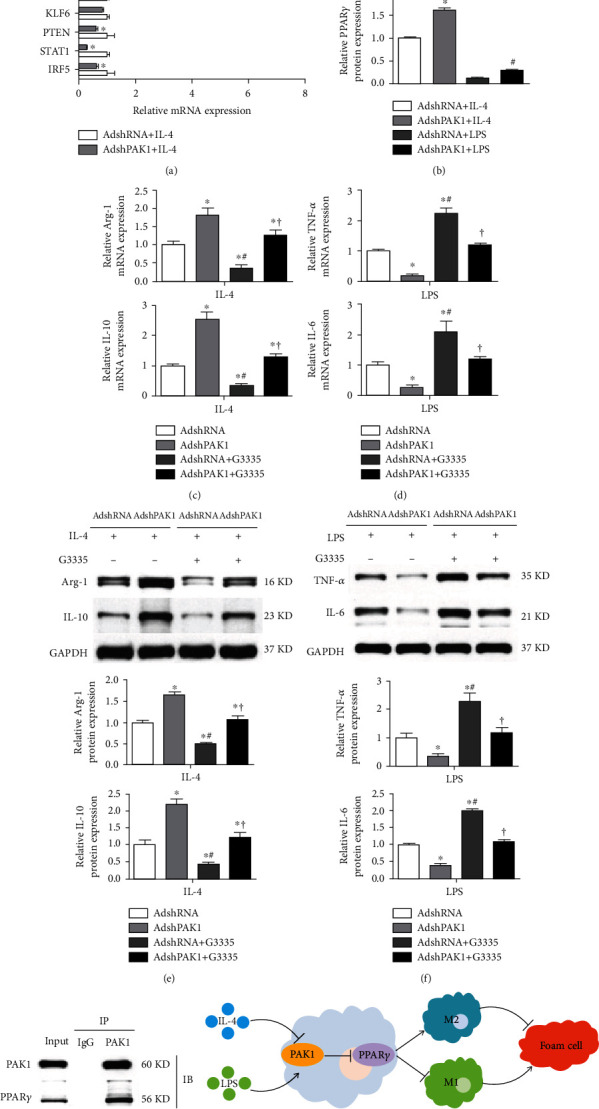
Upregulated PPAR*γ* expression by PAK1 silencing. (a) mRNA levels of the representative regulators for macrophage polarization in BMDMs by PAK1 knockdown with IL-4 stimulation. *n* = 3. ^∗^*P* < 0.05 compared with AdshRNA group. (b) Protein level of PPAR*γ* in BMDMs by PAK1 knockdown with IL-4 or LPS stimulation. *n* = 3. ^∗^*P* < 0.05 or ^#^*P* < 0.05 compared with control group. (c–f). RT-PCR (c, d) and Western blot (e, f) analysis of M2 and M1 marker gene expression in macrophage transfected with AdshRNA or AdshPAK1 and treated with LPS or IL-4, which then cultured with G3335 or control. *n* = 3. ^∗^*P* < 0.05 compared with AdshRNA group; ^#^*P* < 0.05 vs. AdshPAK1 group; ^†^*P* < 0.05 vs. AdshRNA with G3335 group. (g) Immunoblotting with PPAR*γ* or PAK1antibody was performed on co-IP of PAK1 using PAK1 antibody. (h) Schematic diagram of the molecular mechanisms underlying PAK1-regulated macrophage polarization and foam cell. PPAR*γ*: peroxisome proliferator-activated receptor *γ*.

Accumulating evidence has strongly demonstrated that multiple transcription factors are required for management of macrophage polarization [9]. Thus, intensively exploring the underlying mechanism by which PAK1 knockdown alleviated classical M1 macrophages but promoted alternative M2 macrophage activation, we determined changes in the potential aforementioned target genes upon PAK1 silencing. We found that upon PAK1 silencing, PPAR*γ* mRNA levels were increased the most in M2 macrophages (Figure 5(a)), which was confirmed by Western blot analysis (Figure 5(b)). Next, we tested the functional requirement of PPAR*γ* in PAK1-knockdown-mediated macrophage polarization. By using a PPAR*γ*-specific antagonist (G3335), we noticed that PPAR*γ* inactivation blunted the PAK1-knockdown-mediated increased anti-inflammatory M2 phenotype marker expression (Arg-1 and IL-10) and decreased proinflammatory M1 phenotype marker expression (TNF-*α* and IL-6) at both the mRNA (Figures 5(c) and 5(e)) and protein levels (Figures 5(d) and 5(f)). The regulation of PPAR*γ* activity by PAK1 prompted us to ask whether PAK1 could directly interact with PPAR*γ*. Endogenous interaction between PAK1 and PPAR*γ* was identified in BMDMs (Figure 5(g)).

## 4. Discussion

Understanding the switching of macrophages polarization may provide promising targets and novel strategies to protect against the development of atherosclerosis. The current study showed a remarkable enhanced M2 polarized macrophage genes but reduced M1 polarized macrophage marker expression in BMDMs infected with AdshPAK1. Additionally, dramatically decreased foam cell formation was found in PAK1 silencing-induced M2 macrophage activation concomitantly with increased ABCA1 and ABCG1 expression and decreased CD36 and SR-A expression. Mechanistically, the shift in macrophage phenotype acquisition mediated by PAK1 knockdown was largely reversed by PPAR*γ* inactivation. Thus, this evidence establishes the PAK1-PPAR*γ* axis as an attractive therapeutic target for the regulation of macrophage polarization implicated in atherosclerosis (Figure 5(h)).

As a member of the highly conserved family of serine/threonine protein kinases regulated by Ras-related small G-proteins, PAK1 plays diverse roles in cell signaling through catalytic and scaffolding activities, as well as regulation of proliferation and survival pathways, including the MAPK, AKT, Wnt1/*β*-catenin, ER*α*, BAD, and nuclear factor kappa beta (NF-*κ*B) pathways [17, 23, 24]. In addition, PAK1 plays a crucial role in multiple cardiovascular diseases, including cardiac arrhythmias, cardiac contractility dysfunction, hypertrophy, and I/R [19]. Besides, considerable evidences have suggested that PAK1 is involved in inflammation-related diseases. PAK1 promotes the expression of target genes involved in NF-*κ*B signaling [17], while PAK1 deficiency increases Treg cell numbers which are critical for attenuating the local inflammatory response and facilitating a significant improvement in immunopathology during schistosome infection [25]. Moreover, PAK1 interacts with Signal Transducer and Activator of Transcription (STAT3) to form PAK1/STAT3 complex and subsequently regulates the transcription of the IL-6 gene by binding to the IL-6 promoter [26]. Notably, published paper demonstrates that genetic deletion of PAK1 in ApoE-deficient mice leads to reduced atherosclerotic lesion by decreasing IL-6 and MCP-1 levels, whereas the detailed molecule mechanism instead of phenotypic observation remains unexplored. By using PMs and BMDMs isolated from ApoE knockout mice which were utilized as a classical in vitro model for atherosclerosis, we aimed to investigate the potential role of PAK1 in macrophage activation that may contribute to the development of atherosclerosis. Our present data have established that PAK1 may act as an important regulator for macrophage polarization characterized as dramatically increased expression in proinflammatory M1 macrophages but decreased expression in anti-inflammatory M2 macrophages. More importantly, functional study showed that PAK1 silencing suppressed proinflammatory M1 maker expression induced by LPS and restored anti-inflammatory M2 genes subjected to IL-4. Although the present study demonstrated PAK1 as a novel target for regulation of macrophage polarization, the effect of PAK1, especially macrophage-specific PAK1 deficiency, on macrophage activation in vivo should be deeply investigated in the future study.

Substantial evidences have demonstrated that the pathogenesis and evolution of atherosclerotic lesion are significantly influenced by macrophage polarization that M1 or M2 macrophages can, respectively, exert pro- or antiatherogenic functions. Understanding the underlying regulators and mechanisms that are responsible for the dynamic plasticity and distinct functional characteristics of classical M1 macrophage and alternative M2 macrophage activation in the development of atherosclerosis could provide effective strategies to prevent the atherosclerotic lesion development and outcome. Overwhelming suggestive evidences have demonstrated that macrophage polarization is controlled by the interplay between extrinsic factor, intrinsic developmental pathways, and the tissue environment [9]. Stimulated by interferon- (IFN-) *γ* and its receptor with TLR, IL-1R signaling, and TNF induction, macrophages are polarized to M1 phenotype by engaging a set of transcription factors, such as NF-*κ*B, IRF5, STAT1, PTEN, KLF6, and AKT2. By contrast, induced by IL-4, IL-13, and IL-4Ra, the alternative M2 macrophages are polarized with simultaneous activation of key downstream transcription factor that are central to M2 polarization, including STAT6, IRF4, JMJD3, PPAR*δ*, PPAR*γ*, KLF4, and AKT1 [9, 12]. Thus, we continued to elucidate the underlying mechanism by which PAK1 knockdown regulated macrophage polarization. The results showed that PPAR*γ* was the most significantly upregulated gene in macrophages infected with AdshPAK1 upon IL-4 stimulation among those screened targets. Consistently, it has been reported that suppression of PPAR*γ* is responsible for the effect of PAK1 overexpression on NF-*κ*B signaling activation in inflammation and colitis-associated cancer. The nuclear hormone receptor PPAR*γ* has recently emerged as a central switch that determines the pro- or anti-inflammatory potential macrophage *in vitro* and *in vivo* [12, 27]. PPAR*γ* overexpression attenuates the induction of inflammatory gene expression by modulating the downstream transcription factor activity through protein-protein interaction subjected to LPS and IFN*γ* stimulation [28]. By contrast, IL-4- and IL-13-induced differentiation of monocytes into alternative macrophages is enhanced by PPAR*γ*, while secretion of proinflammatory mediators in M1 macrophages is attenuated cocultured with the supernatant obtained from the culture of M2 macrophages with high PPAR*γ* expression [29]. Consistent with these results *in vitro*, selective inactivation of PPAR*γ* in macrophages cause an impairment of alternatively activated M2 macrophages and accelerate diet-induced obesity, insulin resistance, and glucose intolerance, as well as exacerbate atherosclerotic lesion formation in ApoE-deficient mice [12]. In administration of a PPAR*γ*-specific antagonist, it largely reversed the effect of PAK1 silencing on macrophage polarization, while PAK1 can also interact with PPAR*γ*. Considering this evidence, we demonstrated that PAK1 knockdown regulated macrophage polarization partially through the activation of PPAR*γ*.

In addition to its important role in the regulation of macrophage polarization, PPAR*γ* also plays a crucial role in cholesterol transport and foam cell formation. Reduction of SR-A and apoB-48 receptor expression and inhibition of LPL secretion and activity are implicated in attenuated foam cell formation mediated by PPAR*γ* activation [30–32]. Moreover, PPAR*γ* decreases ACAT1 expression to decrease the rate of cholesterol esterification and is positively associated with cholesterol efflux in macrophage through upregulated expression of SR-B1, caveolin-1, ABCA1, and ABCG1 [33–36]. Although foam cell formation has been traditionally linked to the proinflammatory macrophage phenotype, the underlying mechanisms associated with cholesterol loading in reaction to the inflammatory response by macrophages have not yet been defined, and little is known about the propensity of individual polarized macrophage to become foam cells. It has been reported that M4 and Mhem macrophages inhibit the capacity of cholesterol influx but promotes cholesterol efflux depending by increased liver X receptor (LXR) expression, which indicated that M1 or M2 macrophages can act as the main foam cell precursors upon alteration of the key determinates of cholesterol transport [37, 38]. Another important finding in our present study was that increased foam cells were observed in the M1-polarized macrophage population, but fewer foam cells were found in the M2-polarized macrophage population. Importantly, less-significant lipid accumulation was found in AdshPAK1-mediated M2 polarized macrophages induced by IL-4, and this reduction was accompanied by increased expression of markers involved in cholesterol efflux and decreased expression of genes associated with cholesterol influx. Therefore, we suspected that the upregulated PPAR*γ* expression in M2 macrophages mediated by PAK1 knockdown was required for decreased foam cell formation. Collectively, the clearly delineated effect of the “PAK1-PPAR*γ*” axis on macrophage polarization and subsequent foam cell formation indicated the important role of this axis in the development of atherosclerosis.

In summary, our study first demonstrated that PAK1 was a novel regulator of macrophage polarization characterized as positive association between PAK1 expression and proinflammatory macrophage phenotype, increased alternative inflammation-resolving M2 macrophage activation, and more significantly, decreased foam cell formation in PAK1 silencing-induced M2 macrophage activation. Our work provides a promising mechanism and highlights the important role of PAK1 as a promising therapeutic target for atherosclerosis management.

## Figures and Tables

**Table 1:** The primers for real-time PCR.

Primer	Sequence (5′ to 3′)
PAK1-F	GATGTAGCCACAGGGCAGGA
PAK1-R	GAGCCTCCAGCCAAGTATTCC
Arg-1-F	ACACGGCAGTGGCTTTAACC
Arg-1-R	GGCGTTTGCTTAGTTCTGTCTG
Mrc-1-F	TACAGCCGGGAAGACAATAACT
Mrc-1-R	AGGAGTCGGTTAGCAGTATGTTG
IL-10-F	AGTCCTTCAGAGGGGTTCACC
IL-10-R	TTGTCTTGTGGAGCAGGTGTG
KLF4-F	GCCACCCACACTTGTGACTA
KLF4-R	CTGTGTGTTTGCGGTAGTGC
chi3I3-F	TGAAGGAGCCACTGAGGTCT
chi3I3-R	TGAAGGAGCCACTGAGGTCT
Retnla-F	TCCCTCCACTGTAACGAAGAC
Retnla-R	AAGATCCACAGGCAAAGCCA
TNF-*α*-F	TCCCCAAAGGGATGAGAAGTT
TNF-*α*-R	GAGGAGGTTGACTTTCTCCTGG
IL-6-F	CTTCTTGGGACTGATGCTGGT
IL-6-R	CACAACTCTTTTCTCATTTCCACG
iNOs-F	ACATCAGGTCGGCCATCACT
iNOs-R	CAGAGGCAGCACATCAAAGC
IL-1*β*-F	TAATGAAAGACGGCACACCCA
IL-1*β*-R	GTTTCCCAGGAAGACAGGCT
Cox2-F	ATTGCCCTCCCCTCTCTACG
Cox2-R	CGGCTCATGAGTGGAGAACG
MCP-1-F	ATGCAGGTCCCTGTCATG
MCP-1-R	GCTTGAGGTGGTTGTGGA
SR-A-F	TGGAGGAGAGAATCGAAAGCA
SR-A-R	CTGGACTGACGAAATCAAGGAA
CD36-F	GACTGGGACCATTGGTGATGA
CD36-R	AAGGCCATCTCTACCATGCC
ABCA1-F	AGGCACTCAAGCCACTGCTTGT
ABCA1-R	TGCCTCTGCTGTCTAACAGCGT
ABCG1-F	GGTTGCGACATTTGTGGGTC
ABCG1-R	TTCTCGGTCCAAGCCGTAGA
GAPDH-F	TGAAGGGTGGAGCCAAAAG
GAPDH-R	AGTCTTCTGGGTGGCAGTGAT

**Table 2:** Antibody for immunoblot.

Primary antibody	Cat no.	Manufacturer	Sources of species
PAK1	ab223849	Abcam	Rabbit
TNF-a	ab6671	Abcam	Rabbit
IL-6	66146-1-Ig	Proteintech group	Mouse
Arg-1	16001-1-AP	Proteintech group	Rabbit
IL-10	20850-1-AP	Proteintech group	Rabbit
CD36	sc-21772	Santa	Mouse
SR-A	sc-166139	Santa	Mouse
ABCA1	ab66217	Abcam	Mouse
ABCG1	ab52617	Abcam	Rabbit
PPAR*γ*	sc-7273	Santa	Mouse
GAPDH	ab181602	Abcam	Rabbit

## Data Availability

The data used to support the findings of this study are available from the corresponding author upon request.
